# Induction of apoptosis and oxidative stress in estrogen receptor-negative breast cancer, MDA-MB231 cells, by ethanolic mango seed extract

**DOI:** 10.1186/s12906-015-0575-x

**Published:** 2015-03-09

**Authors:** Al-Shwyeh Hussah Abdullah, Abdulkarim Sabo Mohammed, Abdullah Rasedee, Mohamed Elwathig Saeed Mirghani, Mothanna Sadiq Al-Qubaisi

**Affiliations:** Faculty of Food Science and Technology, Universiti Putra Malaysia, 43400 Serdang, Selangor, Malaysia; Faculty of Veterinary Medicine, Universiti Putra Malaysia, 43400 Serdang, Selangor, Malaysia; Institute of Bioscience, Universiti Putra Malaysia, 43400 Serdang, Selangor, Malaysia; Department of Biotechnology Engineering Faculty of Engineering, International Islamic University Malaysia (IIUM), P.O. BOX 10, 50728, Kuala Lumpur, Malaysia

**Keywords:** MDA-MB231 cells, Breast cancer, Apoptosis, Oxidative stress, Mango kernel

## Abstract

**Background:**

In this study, the effect of mango kernel extract in the induction of apoptosis of the breast cancer (MDA-MB-231) cell line was examined. This is an attempt to discover alternatives to current therapeutic regimes in the treatment of breast cancers.

**Methods:**

The pro-apoptotic markers, Bax, cytochrome c, caspases-. -8 and −9, and anti-apoptotic markers, Bcl-2, p53 and glutathione were determined in MDA-MB231 cells treated for 12 and 24 h with mango kernel extract.

**Results:**

The results showed that the extract produced a time- and dose-dependent increases in pro-apoptotic proteins and oxidative stress markers with a corresponding decrease in anti-apoptotic markers.

**Conclusions:**

Based on the findings, mango kernel extract modulates redox balance in MDA-MB-231 breast cancer cells with a tendency for apoptotic cell death. The changes observed in this study may collectively underlie the basis for the cell death induced in MDA-MB-231 breast cancer cells by mango kernel extract. Thus, mango kernel extract has potential to be developed into an antibreast cancer mixture, and hence these results are worth studying further.

## Background

Oxidative stress underlies the pathogenesis of many chronic diseases and their complications [1]. Under normal conditions, unwanted cells are removed from the body through apoptosis, which is responsible for regulating the daily turnover of continuously dividing cells such as the epithelial tissues. In fact oxidative stress is thought to play a role in the apoptotic removal of unwanted cells in the body through induction of death signals [2,3]. This highlights the close association between the signal transduction mechanisms involved in oxidative stress and apoptotic cell death. On the other hand, necrosis, which is also another form of cell death that is commonly seen in cancers as the result of excessive uncontrolled growth often results in extensive damage to normal tissues [4]. Unlike necrosis, apoptosis is governed by several intracellular pathways, which can be influenced or manipulated. Thus, recent studies on therapeutic approaches to cancer have focused on apoptosis as a means to control cancer cell proliferation [5]. The initiator caspases −2, −8, −9 and −10, effector caspases −3, −6, and −7 and the Bcl family of proteins are the main factors in regulation of apoptosis [6,7].

Breast cancer is among the commonest cancer among women, with a significantly high cancer mortality rate. What used to be a health problem for developed countries only, has now become increasing prevalent in developing countries with dire consequences. About 1 million people are diagnosed with breast cancer every year with over 400,000 mortalities [8,9]. The rising cost of management and concerns of side-effects from current therapeutic regimes have necessitated the search for better alternatives for the management and treatment of breast cancers. Among candidates for alternative cancer therapy are phytochemicals from plant bio-resources, which have been studied extensively in recent years for their potential to induce apoptosis in cancers and tumors. Resveratrol, ursolic acid, polyphenols and extracts of *Momordica charantia* have all been reported to induce apoptosis in breast cancer cells [10-12]. Many of these plant derivatives act as antioxidants that regulate oxidative stress and apoptosis. The role of antioxidants in promoting cancer cell death may seem contradictory but studies have now shown that the mechanistic bases of the anticancer effects of some antioxidants is linked to their ability to induce pro-oxidant effects in the cancer cells. Grape seed extract is a clear example of an antioxidant mixture that has been shown to be antioxidant-rich with varying effects on redox balance including induction of oxidative stress and apoptotic pathways in cancer cells that promote cell death [13]. These effects of grape seed extract represent an important mechanism by which antioxidants promote cell death contrary to their perceived effects on promotion of cellular survival.

Mango (*Mangifera indica* L.), is a member of the family *Anacardiaceae*. Mango has become naturalized and adapted to the tropical and subtropical environments [14]. Malaysia, particularly the peninsular area, has several varieties of mangoes, [15]. Mangoes are rich in vitamins, minerals and anti-oxidants [16-18], and their kernels, which are often discarded as waste, are rich in antioxidants. We have recently demonstrated that an ethanolic extract of mango kernel, which is antioxidant-rich, is able to cause death of breast cancer cells but not normal breast cells [19]. Based on our findings and the fact that antioxidants may promote cancer cell death through oxidative stress and apoptosis, we tested the hypothesis that the mango kernel extract induced both oxidative stress and apoptosis in breast cancer cells. Moreover, oxidative stress is implicated in the development and progression of breast cancers [20,21]. Thus, we evaluated the effects of mango kernel extract on oxidative stress and apoptosis in estrogen receptor-negative breast cancer cells, MDA-MB-231 cells.

## Methods

### Preparation of crude extract

Waterlily mango fruits procured from a local market in Kuala Lumpur, Malaysia during the period of June to July, 2012 were identified by a resident botanist (Shamsul Khamis) and a voucher specimen (SK2448/14) was deposited at the Biodiversity unit of the Institute of Bioscience, Universiti Putra Malaysia. Mango samples were manually processed by soaking the kernels in water to remove adhering flesh. The kernels were then dried in an oven at 45°C for 2 d. The dried kernels were ground using Waring blender 7011HS (Osaka Chemical Co. Ltd., Kita-Ku, Osaka, Japan) and stored at 4°C until analysis. Ethanol (95%) was added to the ground kernel at 10:1 (v/w) and the mixture shaken at 200 rpm in an incubator shaker (INNOVA 4000, New Jersey, USA) at 37°C for 24 h. Insoluble materials were filtered and the suspension centrifuged at 4000 rpm (Benchtop Centrifuge Z200A, Labnet International, Inc., Woodbridge, NJ, USA) for 10 mins. The supernatant was dried using 1 L Rotary Evaporator N1001S-WD (Tokyo Rikakikai Co., Ltd., Koishikawa Bunkyo-ku, Tokyo, Japan) and the resulting extract was dissolved in DMSO and stored at −20°C until analyses.

### Cancer cell lines

The estrogen receptor-negative human breast cancer cell line, MDA-MB-231 cells, were obtained from the American Type Culture Collection (ATCC: Rockville, MD, USA) and cultured in DMEM supplemented with 10% FBS and 1% antibiotics (100 U/mL penicillin) in an incubator at 37°C with 5% CO_2_.

### Bcl-2, Bax, p53, and cytochrome c proteins

The determinations of p53, Bax, Bcl-2, and cytochrome C proteins were done using enzyme-linked immune-sorbent assay kits (R&D Systems, Minneapolis, MN, USA) according to the manufacturer’s instructions. The protein levels were determined in 1 × 10^4^ MDA-MB-231 cells treated with 5, 10 and 50 μg/mL mango kernel extract for 12 and 24 h, and the absorbance values were read on a microplate reader. The results were presented as fold change in protein expression relative to the untreated cells.

### Caspase-3, −8 and −9 activities

Commercial kits (Promega, Madison, WI, USA) were used to determine the caspase activities of 1 × 10^4^ MDA-MB-231cells, following the same treatments described above. After treatment, the cells were harvested by centrifugation and the pellets were washed with phosphate-buffered saline (PBS) before lysis in chilled lysis buffer. The mixture was left on ice for 10 min and then centrifuged at 50 × g (Eppendorf Centrifuge 5810R, Hamburg, Germany) at 4°C for 5 min. The resulting supernatant was used for the determination of caspase activities. The results were read on a microplate reader at 405 nm.

### Thiobarbituric acid reactive substances (TBARS)

TBARS are products of lipid peroxidation that are often used to determine the level of oxidative stress. In this study, 1 × 10^4^ treated MDA-MB-231 cells were washed with PBS, harvested, and homogenized in ice-cold 1.15% KCl. The TBARS assay was performed as described previously [22]. The results were expressed as malondialdehyde (MDA) equivalent (nM/mg protein).

### Glutathione (GSH) assay

Treated MDA-MB-231cells (1 × 10^4^) were harvested and lysed for glutathione estimation. The glutathione assay was performed according to a method described previously [22] using the UV–vis spectrophotometer 405 nm and the results expressed as nM/mg cells lysate protein.

### Reactive oxygen species

In this assay, 1 × 10^4^ treated MDA-MB-231 cells were washed with PBS containing 2′,7′-dichlorofluorescein diacetate to oxidize the reactive oxygen species, 2′,7′-dichlorofluorescein diacetate to form dichlorofluorescein [22]. The cells were then lysed in a buffer (50 mM Tris–HCl, 100 mM NaCl, 1 mM CaCl, 1 mM MgCl, 300 mM sucrose, 1% Triton X-100, pH 7.4), and their fluorescence determined in a quartz cuvette at 530 nm at excitation wavelength of 485 nm.

### Statistical analysis

All experiments were performed in triplicates, and the data were expressed as the mean ± standard deviation. The data were analyzed using Minitab statistical software (Minitab Inc, State College, PA, USA). One-way analysis of variance followed by Tukey’s post hoc analysis was done to determine statistical significance (*P <* 0.05).

## Results and discussion

### BAX, Bcl-2 and cytochrome c 2 levels

**Figure 1:**
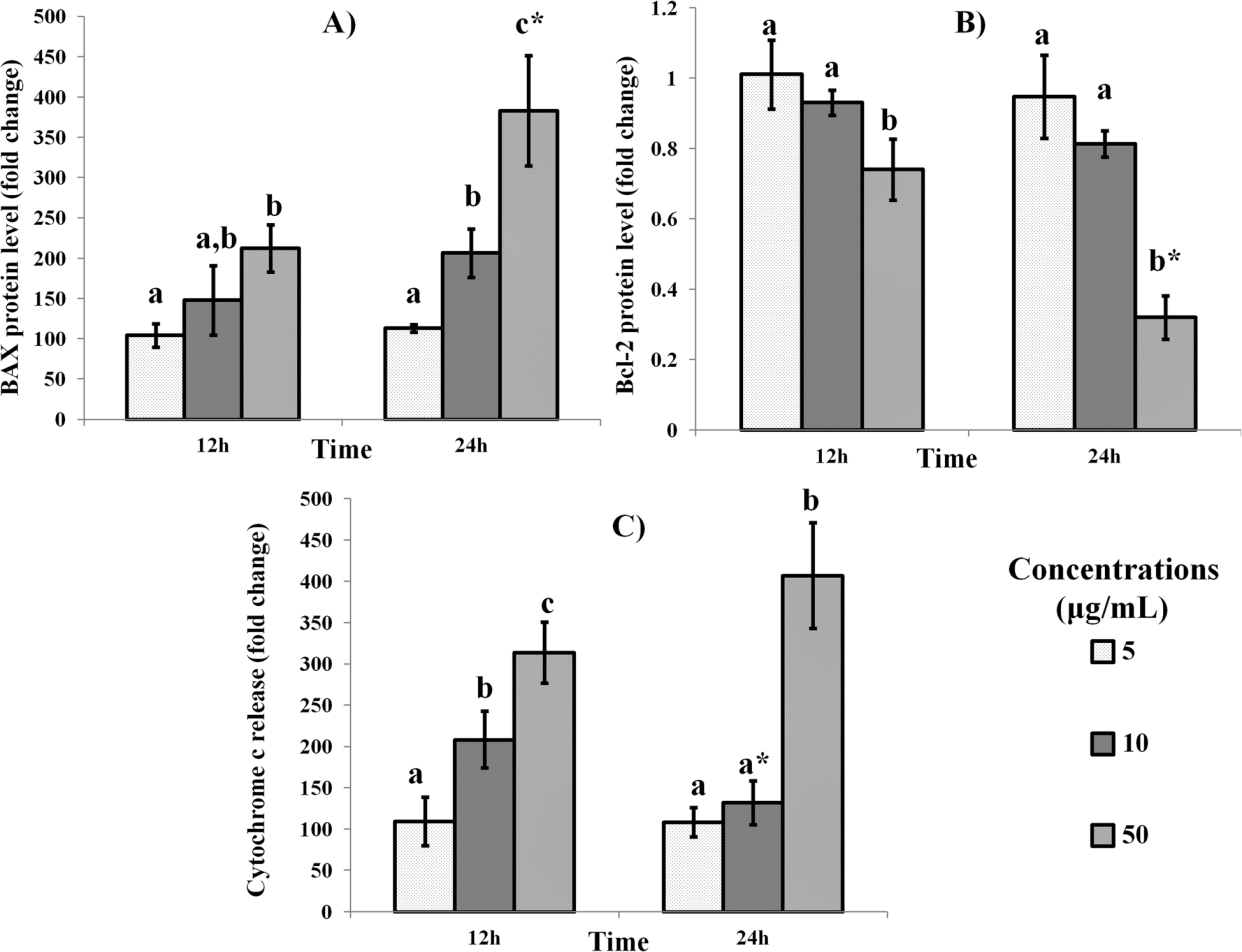
**Effect of Mango kernel extract on (A) Bax, (B) Bcl-2 and (C) cytochrome c levels in MDA-MB-231 cells.** Treatment with 5, 10 and 50 μg/mL of mango kernel extract over 12 and 24 h produced progressively higher levels of Bax and cytochrome c and lower levels of Bcl-2 in a dose-dependent manner. Bars with different letters for any time period of an assay indicate significant difference. *Denote significant difference at 24 h when compared to the same concentration at 12 h.

Apoptosis is a complex process involving several pathways and factors including Bax, Bcl-2 and cytochrome c [4]. Bax promotes apoptosis while BCL-2 is antiapoptotic. During the initiation of apoptosis, cytochrome c released from the mitochondria activates the proapoptotic caspase-9, which ultimately causes apoptotic cell death [4-6]. In this study, treatment of MDA-MB-231 cells with mango kernel extract caused progressive dose-dependent increase in the levels of both Bax and cytochrome c and decrease in BcL-2 over 24 h (Figure 1). Our earlier report on induction of cancer cell death in MDA-MB-231 cells but not normal breast cells [19] and the present findings suggest that the killing of the cancer cells by mango kernel extract may be through the stimulation of proapototic and inhibition of antiapoptotic proteins.

### Caspase-3, −8 and −9 activities

**Figure 2:**
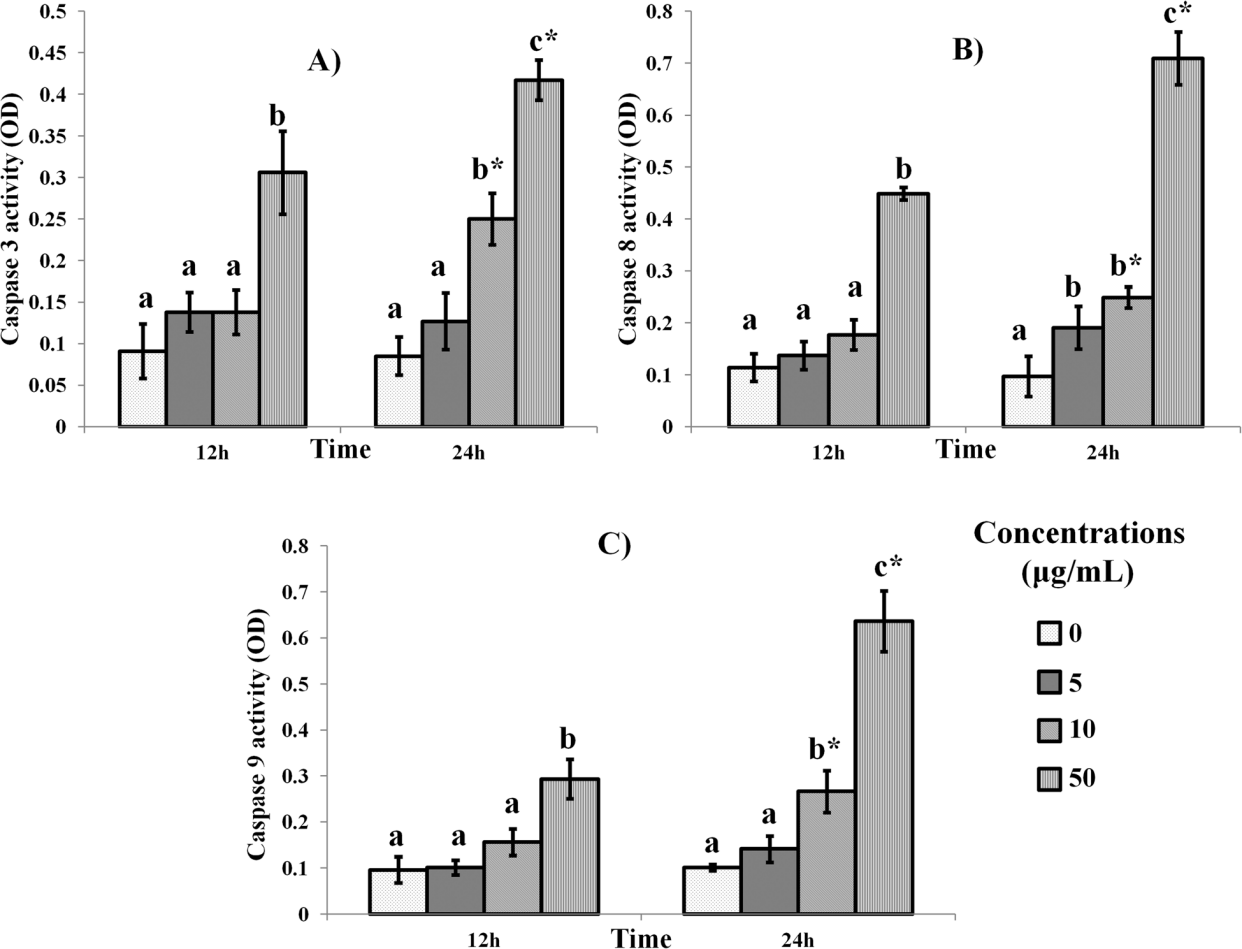
**Effect of Mango kernel extract on (A) caspases-3, (B) caspase-8 and, (C) caspase-9 activities in MDA-MB-231 cells.** Treatment with 5, 10 and 50 μg/mL of mango kernel extract over 12 and 24 h produced progressively higher levels of all caspases in a dose-dependent manner. Bars with different letters for any time period of the assay indicate significant difference. *Denote significant difference at 24 h when compared to the same concentration at 12 h.

Caspases −3, −8 and −9 are also key regulators of the apoptotic pathways [4,5]. Mango kernel extract showed dose-dependent effects on the activities of the caspases in MDA-MD-231 cancer cells (Figure 2). At 24 h, the activities of all caspases were highest with 50 μg/mL extract treatment. At this concentration the caspase activities were significantly (*P < 0.05*) higher than at either 5 or 10 μg/mL extract. The increases in activities of caspases-8 and −9, which are regulators of the extrinsic and intrinsic pathway of apoptosis, respectively [4,5] indicated that the mango kernel extract produced its effects through activation of both pathways of apoptosis, perhaps suggesting the efficaciousness of the extract in inducing apoptosis of MDA-MB-231 cells.

### Oxidative stress markers

**Figure 3:**
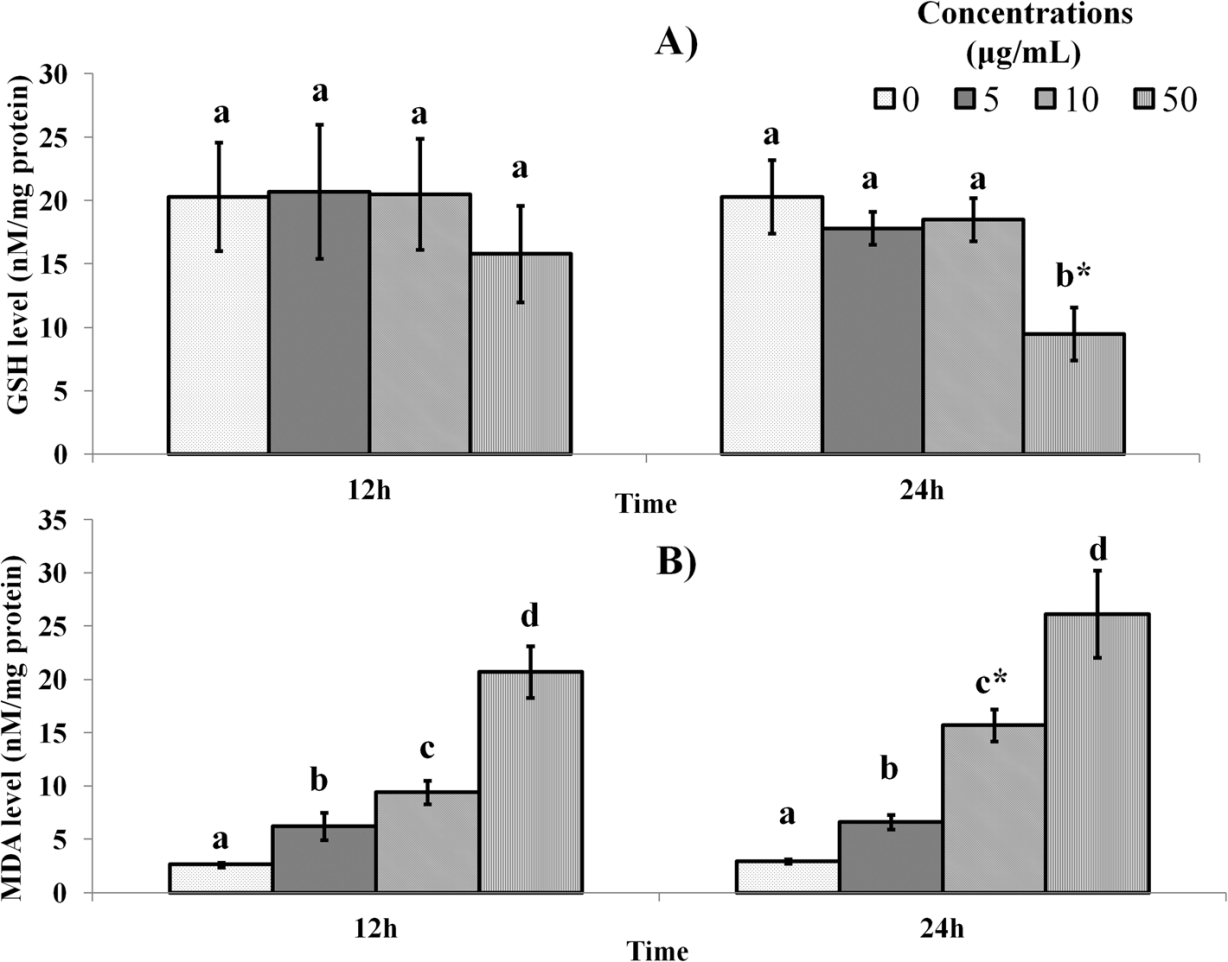
**Effect of mango kernel extract on (A) glutathione (GSH) and, (B) malondialdehyde (MDA) levels in MDA-MB-231 cells.** Only 50 μg/mL extract decreased the level of GSH significantly (P < 0.05) significantly after 24 h, while there was a dose-dependent increase in MDA levels after 12 and 24 h. Bars with different letters for any time period of an assay indicate significant difference. *Denote significant difference at 24 h when compared to the same concentration at 12 h.

Depletion of GSH is an indicator of apoptosis. Moreover, low cellular GSH content is often associated with cell death probably as a result of imbalance in redox states of cells and loss of the protection conferred by this antioxidant [23,24]. In this study, the GSH in the treated MDA-MB-231 cells decreased significantly (*P < 0.05*) only after treatment with 50 μg/mL of mango kernel extract after 24 h (Figure 3A). Since previous reports have suggested that oxidative stress signals are initiators of apoptosis [2,3], the decrease in the antioxidant GSH was likely an indicator that the redox balance in the breast cancer cells was shifted in favour of apoptosis. Moreover, increased MDA levels in the MDA-MB-231 breast cancer (*P < 0.05*) due to mango kernel extract treatment (Figure 3B), were a pointer to increased cellular oxidative stress, which may in turn have promoted apoptosis

**Figure 4:**
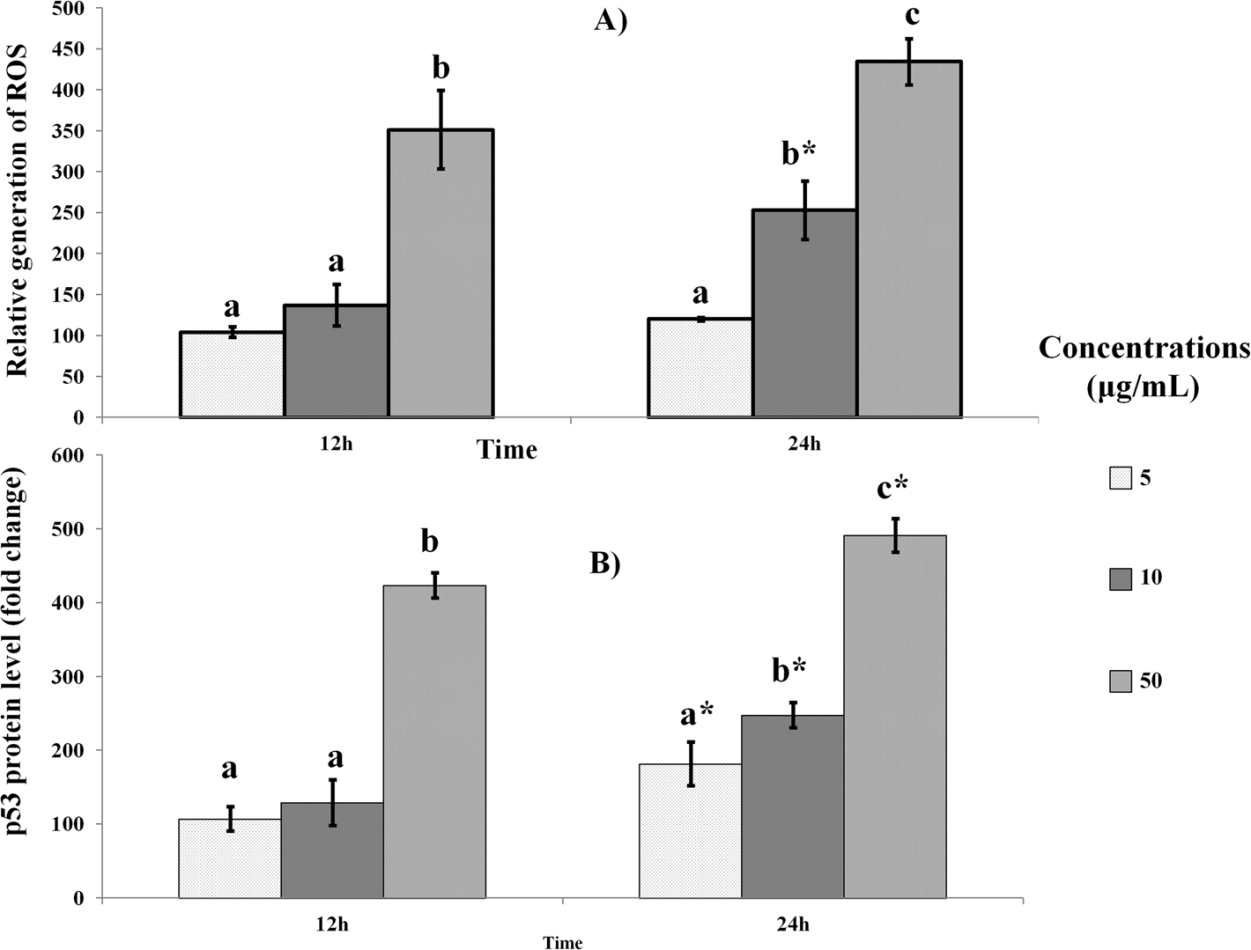
**Effect of mango kernel extract on (A) generation of reactive oxygene species (ROS) and, (B) p53 level in MDA-MB-231 cells.** Treatment with extract produced time- and dose-dependent increases in ROS and p53. Bars with different letters for any time period of an assay indicate significant difference. *Denote significant difference at 24 h when compared to the same concentration at 12 h.

The generation of ROS by mango kernel extract-treated MDA-MB-231 cells was shown to be time- and dose-dependent (Figure 4A), which parallels the increase in MDA levels in these cells. This further suggests that mango kernel extract induced cellular oxidative stress, which may have also promoted apoptosis through activation of Bax and cytochrome c release. At the same time, p53 was shown to increase in the treated cells in time- and dose-dependent manner (Figure 4B). p53 is a tumor suppressor that regulates cell survival, and its decreased expression was reported to occur in cancers [25,26]. In fact, it has been suggested that the down-regulation of Bcl-2 is associated with p53-induced apoptosis [26], as may be the case in the current study. Additionally, oxidative stress has also been reported to activate p53 [27], which the increases in MDA and ROS in this study are indicative of. Hence, it is likely that mango kernel extract induced apoptosis secondary to oxidative stress or they have both been consequences of the extract.

In aggregate, the data from this study suggests that mango kernel extract is able to modulate the redox balance in estrogen-negative breast cancer cells with consequent increases in oxidative stress and apoptosis. Increases in caspase activities, Bax and cytochrome c and decrease in Bcl-2 are evidences that treatment with the extract favors apoptosis rather than survival of cancer cells, while increases in MDA and ROS suggest induction of oxidative stress in the cancer cells. It can be recalled that antioxidants may promote cancer cell death through modulating redox balance and promoting apoptotic cell death through various mechanism [13], similar to what we have demonstrated in this study. Our results show that despite the rich antioxidant content of ethanolic extract of mango kernel extract [19], which may be expected to promote cell survive, the exert in fact causes death of breast cancer cells. Due to concerns of side-effects and the rising cost of cancer management and control with pharmacological agents, non-toxic natural products have received more attention as alternatives. Our results show that mango kernel extract may be a promising and cheaper source of natural remedy for breast cancer. The cheaper alternative therapeutic compounds are especially essential for patients in developing countries with low income [27,28].

## Conclusions

This study showed that mango kernel extract was able to increase expression of markers of apoptosis, Bax, cytochrome c, MDA, p53, ROS and caspases in breast cancer cells. Concomitantly, the extract also decreased pro-survival factors, GSH and Bcl-2 in the cancer cells, providing additional evidence that the mango kernel extract has anticancer properties. These findings suggest that mango kernel extract has potential to be developed into a therapeutic mixture for treatment of breast cancers.
